# The optimal cutoff of atrial high‐rate episodes for neurological events in patients with dual chamber permanent pacemakers

**DOI:** 10.1002/clc.23626

**Published:** 2021-05-18

**Authors:** Wei‐Da Lu, Ju‐Yi Chen

**Affiliations:** ^1^ Department of Internal Medicine National Cheng Kung University Hospital, College of Medicine, National Cheng Kung University Tainan Taiwan

**Keywords:** atrial fibrillation, atrial high‐rate episodes, dual chamber pacemakers, neurological events

## Abstract

**Background:**

Patients with atrial high‐rate episode (AHRE) are at higher risk of neurological events. This study aimed to identify the optimal cutoff threshold for AHRE duration in patients with dual chamber permanent pacemakers (PPM) without prior atrial fibrillation.

**Methods:**

We included 355 consecutive patients receiving dual chamber pacemaker implantation. Primary outcome was composite endpoint of subsequent neurological events after various AHRE durations. AHRE was defined as >175 bpm (MEDTRONIC) or > 200 bpm (BIOTRONIK) for longer than 30 s. Cox regression analysis with time‐dependent covariates was conducted.

**Results:**

The mean age of included patients was 75.6 ± 11.3 years. Among 355 included patients, some had multiple AHREs; 125 patients (35.2%) developed AHRE ≥2 min, 107 (30.1%) had ≥5 min, 55 (15.5%) had ≥6 h, and 37 (10.4%) had ≥24 h. The mean follow‐up was 42.1 ± 31.2 months. During follow‐up, 19 neurological events occurred. After adjustment for CHA_2_DS_2_‐VASc score and device type, multivariate Cox regression analysis indicated AHRE ≥2 min (HR 13.605, 95% CI 3.010–61.498), and AHRE ≥5 min (HR 5.819, 95% CI 2.056–16.470) were significantly associated with neurological events. Hence, the optimal AHRE cutoff value was 2 min with the highest Youden index (sensitivity, 89.5%; specificity, 67.8%; AUC, 0.823, 95% CI, 0.763–0.884; p < 0.001).

**Conclusions:**

Patients with dual chamber PPM who develop AHRE have increased risk of neurological events. Comprehensive assessment of the risks and benefits of prescribing anticoagulants should be considered in PPM patients with AHRE ≥2 min.

## 1 INTRODUCTION

Atrial fibrillation (AF), despite good progress with its management, remains a common arrhythmia encountered in clinical practice and is a major cause of systemic thromboembolic diseases, such as stroke and systemic embolism.^1^ AF is diagnosed by 12‐lead electrocardiography and may be transient and asymptomatic, leading to difficulty in its detection. The use of cardiac implantable electronic devices (CIEDs) is increased because of the technical ability to monitor long‐term atrial rhythm.

Recently, subclinical AF (SCAF), also called atrial high‐rate episode (AHRE), is detected by CIEDs.^2^ Even in asymptomatic patients, AHRE has been shown to be associated with an elevated risk of neurological events, including stroke and transient ischemic attacks^3^; however, this risk seems to be lower than in patients with diagnosed AF.^4^ The optimal burden or cutoff value for AHRE contributing increasing risk of neurological events remains controversial. AHRE lasting ≥30 s,^5^ ≥ 5 min,^6^ ≥ 6 min,^2^ ≥ 6 h,^7^ and ≥ 24 h^8^ have been shown to be related to an increased risk of systemic thromboembolic events. Currently, CIEDs should be interrogated on a regular basis for AHRE.^1^ Patients with AHRE should undergo further assessment for systemic thromboembolic risk factors and for overt AF, including ECG monitoring. The recommended AHRE duration, for patients without known AF, as per 2016 guidelines, is >180 bpm lasting longer than 5–6 min, as detected by an implanted device.^1^ Hence, we examined the associations between a range of cutoff durations of AHRE and the incidence rates of neurological events in Taiwanese patients with dual chamber permanent pacemakers (PPM).

## 2 METHODS

### 2.1 Study participants

We recruited patients older than 18 years old with dual chamber PPM (MEDTRONIC or BIOTRONIK) treated in the Cardiology Department of National Cheng Kung University Hospital, from January 2015 to August 2019. The protocol for this cohort study was reviewed and approved by the ethics committee of National Cheng Kung University Hospital (B‐ER‐108‐278), and was conducted according to the guidelines of the International Conference on Harmonization for Good Clinical Practice. We ensure that we have specified whether all data were fully anonymized before we accessed them and the ethics committee waived the requirement for informed consent.

### 2.2 Data collection and definitions

Patients' medical history, comorbidities, and echocardiographic parameters were collected from chart records for retrospective evaluation. Diabetes mellitus was defined by the presence of symptoms and a random plasma glucose concentration ≥ 200 mg/dl, fasting plasma glucose concentration ≥ 126 mg/dl, 2 h plasma glucose concentration ≥ 200 mg/dlL, from a 75 g oral glucose tolerance test, or taking medication for diabetes mellitus.^9^ Hypertension was defined as in‐office systolic blood pressure (SBP) ≥ 140 mmHg and/or diastolic BP (DBP) ≥ 90 mmHg or taking antihypertensive medication.^10^ Dyslipidemia was defined as low‐density lipoprotein ≥140 mg/dl, high‐density lipoprotein <40 mg/dl, triglycerides ≥150 mg/dl, or taking medication for dyslipidemia.^11^ Chronic kidney disease was defined as an estimated glomerular filtration rate (eGFR) < 60 ml/min/1.73 m^2^.^12^ Neurological events were defined as either ischemic stroke or transient ischemic attack (TIA), definitively diagnosed by an experienced neurologist. A TIA was defined as a transient episode of neurologic dysfunction caused by focal brain, spinal cord, or retinal ischemia, without acute infarction.^13^ Ischemic stroke was defined as acute focal or global disturbance of cerebral function due to vascular dysfunction, which lasted longer than 24 h or resulted in death.^14^ AHRE were extracted from the devices via telemetry at each office visit every 3–6 months. AHRE electrograms were reviewed by at least one experienced electrophysiologist, who carefully considered the possibility that AHRE included lead noise or artifact, far‐field R‐waves, or paroxysmal supraventricular tachycardia and visually confirmed AF in the detected AHRE (Supplement Figure 1). Atrial sensitivity was programmed to 0.3 mV with bipolar sensing of MEDTRONIC and 0.2 mV with bipolar sensing of BIOTRONIK.

The primary endpoint for this study was the occurrence of neurological events after the date of implantation of a pacemaker. AHRE were defined as atrial rate > 175 bpm (MEDTRONIC) or > 200 bpm (BIOTRONIK) and lasting for at least 30 s of atrial tachyarrhythmia recorded by the devices on any day during the study period. AHREs were classified into six duration groups: ≥ 30 s, ≥ 1 min, ≥ 2 min, ≥ 5 min, ≥ 6 h and ≥ 24 h, to evaluate the cutoff threshold for neurological events. If the patient had multiple AHREs, the longest AHRE duration was used for analysis. Then, if the patient's longest AHRE duration was 6 min, this patient would be counted in AHRE ≥  30 s, AHRE ≥ 1 min, AHRE ≥ 2 min, and AHRE ≥ 5 min.

### 2.3 Statistical analysis

Among baseline characteristics, categorical variables are presented as percentages. Continuous variables are presented as means and standard deviations. Chi‐square test or Fisher's exact test was used for categorical variables, and the two‐sample student's *t*‐test for continuous variables. Factors with significant differences (p < 0.10) in univariate analysis were then entered into multivariate Cox regression analysis. Cox regression analysis was used to identify variables associated with AHRE occurrence, reported as hazard ratios with 95% confidence intervals (CI). Indicators of AHRE ≥ 30 s, ≥ 1 min, ≥ 2 min, ≥ 5 min, ≥ 6 h, and ≥ 24 h were determined separately as time‐dependent covariates in multivariate Cox proportional hazards regression, and survival curves were generated for patients without neurological events. The receiver‐operating characteristic (ROC) area under the curve (AUC) from AHRE and their associated 95% confidence intervals (CI) were investigated for association with future neurological events. The optimal cutoff values were chosen based on the results of ROC curve analysis with the highest Youden index and used to evaluate the associated values of AHRE, in minutes, for determining end points. For all comparisons, p < 0.05 was considered statistically significant. All data were analyzed using SPSS statistical package version 23.0 (SPSS Inc. Chicago, IL, USA).

## 3 RESULTS

### 3.1 Patient characteristics

From January 01, 2014 to August 31, 2019, a total of 498 consecutive patients receiving dual chamber PPMs at our hospital were initially recruited. Patients were excluded due to loss of follow‐up (10), or inadequate or missing data (3). Patients with a history of atrial fibrillation (130) were also excluded. After exclusions, 355 patients were included in this retrospective study.

Mean follow‐up was 42.1 ± 31.2 months after the implantation of a dual chamber PPM. Table 1 shows baseline characteristics and demographic data of all patients based on the occurrence of AHRE ≥30 s, ≥ 1 min, ≥ 2 min, ≥ 5 min, ≥ 6 h or ≥ 24 h. Mean age was 75.6 ± 11.3 years; 42.8% were women. The most common indication for dual chamber permanent pacemaker implantation was sick sinus syndrome (66.2%), followed by atrioventricular block (33.8%). High levels of hypertension (92.4%) and hyperlipidemia (90.4%) suggest a relatively high risk of neurological events for the entire study cohort (Table 1). During follow‐up, 162 (45.6%) patients developed AHRE ≥30 s, 145 (40.8%) developed AHRE ≥1 min, 125 (35.2%) developed AHRE ≥2 min, 107 (30.1%) developed AHRE ≥5 min, 55 (15.5%) developed AHRE ≥6 h, and 37 (10.4%) patients developed AHRE ≥24 h. Demographics, temporal data of the neurologic events, and type and incidence of neurological events are presented in Tables 2 and 3. Follow‐up was comprised of 1245.84 patient‐years of observation. The total number of neurological events that occurred was 19 (IR 1.53%/year, 95% CI 0.98–2.38), which includes TIA (total number 12, IR 0.96%/year, 95% CI 0.55–1.69) and ischemic stroke (total number 7, IR 0.56%/year, 95% CI 0.27–1.18). Incidence of atrial fibrillation and neurological events, stratified by AHRE durations, are shown in Tables 4 and 5. All patients with subsequent documented atrial fibrillation received anticoagulant therapy.

**TABLE 1:** Baseline characteristics of the overall study group

Variables	All patients (*n* = 355)	Neurological event		Univariate p valve
		Yes (*N* = 19)	No (*N* = 336)	
Age (years)	75.6 ± 11.3	77.3 ± 9.4	75.5 ± 11.4	0.502
Gender				0.057
Male	203 (57.2%)	15 (78.9%)	188 (56.0%)	
Female	152 (42.8%)	4 (21.1%)	148 (44.0%)	
BMI (kg/m^2^)	24.4 ± 2.3	24.3 ± 2.1	24.5 ± 2.3	0.795
Device				0.051
MEDTRONIC	220 (62.0%)	16 (84.2%)	204 (60.7%)	
BIOTRONIK	135 (38.0%)	3 (15.8%)	132 (39.3%)	
Primary indication				0.223
Sinus node dysfunction	235 (66.2%)	16 (84.2%)	219 (65.2%)	
Atrioventricular block	120 (33.8%)	3 (15.8%)	117 (34.8%)	
CHA_2_DS_2_‐VASc score	3.2 ± 1.3	3.8 ± 1.4	3.2 ± 1.3	0.056
HAS‐BLED	2.2 ± 1.2	2.6 ± 0.7	2.2 ± 1.2	0.165
Hypertension	328 (92.4%)	19 (100.0%)	309 (92.0%)	0.381
Diabetes mellitus	185 (52.1%)	14 (73.7%)	171 (50.9%)	0.061
Hyperlipidemia	321 (90.4%)	19 (100%)	302 (89.9%)	0.236
Prior stroke	14 (3.9%)	4 (21.1%)	10 (3.0%)	0.004
Prior myocardial infarction	72 (20.3%)	4 (21.1%)	68 (20.2%)	1.000
Heart failure				0.322
Preserved EF	28 (7.9%)	2 (10.5%)	26 (7.7%)	
Reduced EF	40 (11.3%)	4 (21.1%)	36 (10.7%)	
Chronic kidney disease	18 (5.1%)	8 (42.1%)	125 (37.2%)	0.668
Chronic liver disease	133 (37.5%)	2 (10.5%)	16 (4.8%)	0.249
Echo parameters				
LVEF (%)	66.1 ± 12.8	63.2 ± 15.4	66.3 ± 12.7	0.308
Mitral E/e′	12.4 ± 5.3	11.8 ± 4.8	12.4 ± 5.4	0.608
LA diameter (cm)	3.7 ± 0.6	3.8 ± 0.6	3.7 ± 0.6	0.297
RV systolic function (s', m/s)	12.6 ± 1.7	12.5 ± 2.0	12.6 ± 1.7	0.715
Drug prescribed at baseline				
Antiplatelets	128 (36.1%)	12 (63.2%)	116 (34.5%)	0.011
Anticoagulants	32 (9.0%)	2 (10.5%)	30 (8.9%)	0.685
Beta blockers	96 (27.0%)	6 (31.6%)	90 (26.8%)	0.647
Amiodarone	44 (12.4%)	3 (15.8%)	41 (12.2%)	0.717
Propafenone	15 (4.2%)	0 (0%)	15 (4.5%)	1.000
Digoxin	4 (1.1%)	0 (0%)	4 (1.2%)	1.000
non‐DHP CCBs	12 (3.4%)	0 (0%)	12 (3.6%)	1.000
RAAS inhibitors	138 (38.9%)	7 (36.8%)	131 (39.0%)	0.844
Diuretics	57 (16.1%)	5 (26.3%)	52 (15.5%)	0.211
Statins	121 (34.1%)	7 (36.8%)	114 (33.9%)	0.794
Metformin	57 (16.1%)	4 (21.1%)	53 (15.8%)	0.523
SGLT2 inhibitors	4 (1.1%)	0 (0%)	4 (1.2%)	1.000
Follow duration (months)	42.1 ± 31.2	29.5 ± 27.8	42.8 ± 31.3	0.070
Follow times	5.8 ± 4.4	4.1 ± 3.5	5.9 ± 4.4	0.071
AHRE duration**≥**30 s	162 (45.6%)	19 (100%)	143 (42.6%)	<0.001
AHRE duration **≥** 1 min	145 (40.8%)	19 (100%)	126 (37.5%)	<0.001
AHRE duration **≥** 2 min	125 (35.2%)	17 (89.5%)	108 (32.1%)	<0.001
AHRE duration **≥** 5 min	107 (30.1%)	14 (73.7%)	93 (27.7%)	<0.001
AHRE duration **≥** 6 h	55 (15.5%)	6 (31.6%)	49 (14.6%)	0.046
AHRE duration **≥** 24 h	37 (10.4%)	5 (26.3%)	32 (9.5%)	0.020

*Note*: Data are presented as mean ± SD or *n* (%).

Abbreviations: AF, atrial fibrillation; AHRE, atrial high‐rate episodes; BMI, body mass index; EF, ejection fraction; LA, left atrium; LVEF, left ventricular ejection fraction; RV, right ventricle; non‐DHP CCBs, non‐dihydropyridine calcium channel blockers; RAAS, renin‐angiotensin‐aldosterone system; SGLT2, sodium glucose co‐transporters 2.

**TABLE 2:** Demographic data in all patients with ischemic stroke or TIA

Number	Event	Age	Sex	Indication	CHA2DS2‐VASc score	Time from PPM to the first detection of AHRE (month)	Time from the first detection of AHRE to neurological events (month)	The longest AHRE (e.g., in hours) prior to neurologic events (hour)	Anti‐platelet	Anticoagulant
1	TIA	68	M	SSS	6	1	2	6.00	Y	N
2	TIA	83	M	SSS	7	6	6	816.00	N	N
3	TIA	74	F	SSS	4	8	28	.50	N	N
4	TIA	64	M	SSS	3	4	2	.06	Y	N
5	IS	89	M	AVB	4	3	5	.05	Y	N
6	TIA	57	F	AVB	3	2	25	.03	N	N
7	TIA	86	M	SSS	3	3	93	.02	Y	N
8	TIA	83	M	AVB	3	2	10	2256.00	N	Y
9	TIA	84	M	SSS	5	2	9	3600.00	N	N
10	IS	71	M	SSS	4	1	10	.06	Y	N
11	TIA	82	M	SSS	3	1	2	29520.00	Y	N
12	IS	69	M	SSS	2	1	1	.15	Y	N
13	TIA	76	F	SSS	6	1	2	1.00	Y	N
14	IS	94	F	SSS	4	3	44	2.00	Y	N
15	TIA	79	M	SSS	4	2	23	.24	Y	N
16	IS	68	M	SSS	2	1	24	10.00	N	N
17	IS	78	M	SSS	3	2	60	2.00	Y	N
18	IS	86	M	SSS	3	4	1	504.00	Y	N
19	TIA	77	M	SSS	3	3	2	3.00	N	Y

Abbreviations: AHRE, atrial high‐rate episodes; AVB, atrioventricular block; F, female; IS, ischemia stroke; M, male; N, no; PPM, permanent pacemaker; SSS, sick sinus syndrome; TIA, transient ischemic attack; Y, yes.

**TABLE 3:** Type and incidence of neurological events in the cohort

Types of neurological events	Number	Incidence rate (100 patient‐years)	CI 95%	Time to event (months)	Age (years)	Gender (female)	Prior stroke	Antiplatelets	Anticoagulant
TIA	12 (3.4%)	0.96	0.55–1.69	21.7 ± 25.7 (2–96)	76.1 ± 8.9	3 (25%)	3 (25%)	6 (50%)	2 (16.7%)
Ischemic stroke	7 (2.0%)	0.56	0.27–1.18	23.7 ± 22.6 (1–62)	79.3 ± 10.5	1(14.3%)	1(14.3%)	6 (85.7%)	0 (0%)
Total events	19	1.53	0.98–2.38						

*Note*: Data are presented as mean ± SD or *n* (%).

Abbreviations: AHRE, atrial high‐rate episodes; TIA, transient ischemic attack.

**TABLE 4:** Incidence of atrial fibrillation among patients with different AHRE durations

AHRE durations	Number	Incidence rate (100 patient‐years)	CI 95%
All patient	32 (9.0%)	2.57%	1.82–3.62
**≥** 30 s	26 (16.0%)	4.43%	3.05–6.46
**≥** 1min	26 (17.9%)	4.89%	3.36–7.11
**≥** 2 min	23 (18.4%)	4.97%	3.33–7.41
**≥** 5 min	22 (20.6%)	5.43%	3.62–8.15
**≥** 6 h	14 (25.5%)	6.95%	4.19–11.51
**≥** 24 h	13 (35.1%)	10.77%	6.44–17.99

Abbreviations: AHRE, atrial high‐rate episodes.

**TABLE 5:** Incidence of neurological events among patients with different AHRE durations

AHRE durations	Number	Incidence rate (100 patient‐years)	CI 95%
**≥** 30 s	19 (11.7%)	3.24%	2.08–5.04
**≥** 1min	19 (13.1%)	3.57%	2.30–5.55
**≥** 2 min	17 (13.6%)	3.68%	2.31–5.86
**≥** 5 min	14 (13.1%)	3.46%	2.07–5.78
**≥** 6 h	6 (10.9%)	3.98%	1.35–6.55
**≥** 24 h	5 (13.5%)	4.14%	1.76–9.77

Abbreviations: AHRE, atrial high‐rate episode.

### 3.2 Univariate analysis and multivariate Cox regression analysis of associations between duration of AHRE and neurological events in all patients

Univariate analysis found an association of gender, device type, CHA_2_DS_2_‐VASc score, and diabetes mellitus, with neurological events, to be only of borderline significance. Prior stroke, AHRE duration ≥30 s, AHRE duration ≥1 min, AHRE duration ≥2 min, and AHRE duration ≥5 min, ≥ 6 h and ≥ 24 h, were significantly associated with neurological events occurrence in all patients (Table 1). When CHA_2_DS_2_‐VASc score and device type were confounders, AHRE ≥2 min (HR 13.605, 95% CI 3.010–61.498, p = 0.001) and AHRE ≥5 min (HR 5.819, 95% CI (2.056–16.470, p = 0.001) were still independently associated with neurological events (Table 6). Multivariate Cox regression analysis revealed that, except for prior stroke, AHRE ≥2 min (HR 13.406, 95% CI 2.959–60.743, p = 0.001), AHRE ≥5 min (HR 5.725, 95% CI 1.960–16.720, p = 0.001), and AHRE ≥24 h (HR 2.950, 95% CI 1.008–8.634, p = 0.048) were all significantly associated with neurological events (Supplementary Table 1). However, AHRE ≥6 h (HR 2.401, 95% CI 0.862–6.687, p = 0.094) was not significantly associated with neurological events (Supplementary Table 1).

**TABLE 6:** Multivariate Cox regression for neurological events

Variables	Multivariate Cox regression																	
	Model 1			Model 2			Model 3			Model 4			Model 5			Model 6		
	HR	95%CI	p	HR	95%CI	p	HR	95%CI	p	HR	95%CI	p	HR	95%CI	p	HR	95%CI	p
CHA_2_DS_2_‐VASc score	1.669	1.144–2.433	0.008	1.614	1.114–2.339	0.029	1.587	1.093–2.305	0.015	1.669	1.144–2.433	0.008	1.614	1.114–2.339	0.029	1.587	1.093–2.305	0.015
Device (Medtronic)	1.131	0.305–4.188	0.854	1.075	0.102–1.298	0.119	0.682	0.181–2.571	0.572	0.399	0.112–1.27	0.158	0.306	0.086–1.083	0.066	0.317	0.089–1.134	0.077
AHRE duration **≥** 30 s	240 426	0.000–1969	0.905															
AHRE duration **≥** 1 min				300 138	0.000–3201	0.905												
AHRE duration **≥** 2 min							13.605	3.010–61.498	0.001									
AHRE duration **≥**5 min										5.819	2.056–16.470	0.001						
AHRE duration **≥**6 h													2.031	0.7575.454	0.160			
AHRE duration**≥**24 h																2.277	0.791–6.553	0.127

*Note*: Data are presented as mean ± SD or *n* (%).

Abbreviations: AHRE, atrial high‐rate episodes.

### 3.3 ROC‐AUC determination of AHRE cutoff values for association with future neurological events

**FIGURE 1:**
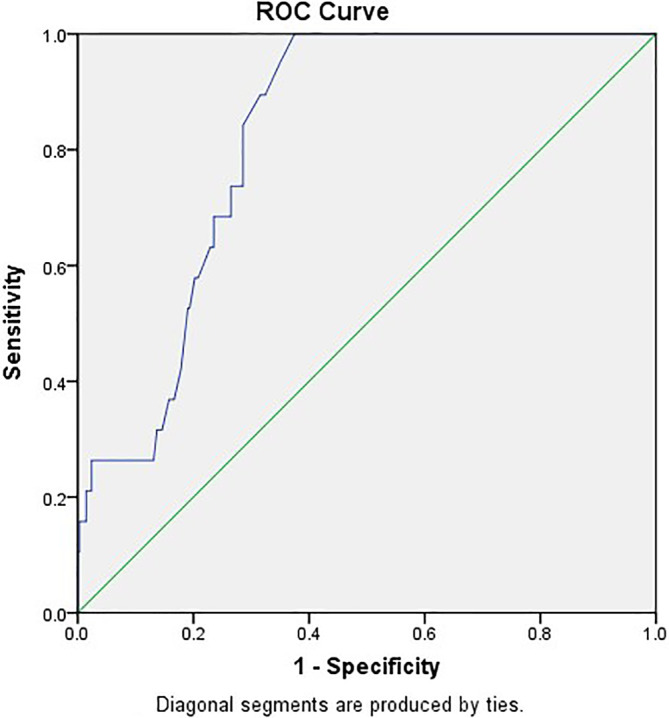
Atrial high‐rate episodes (minutes): cutoff value, 2 min; sensitivity, 89.5%; specificity, 67.8%; AUC, 0.823; 95% CI, 0.763–0.884; p < 0.001

**FIGURE 2:**
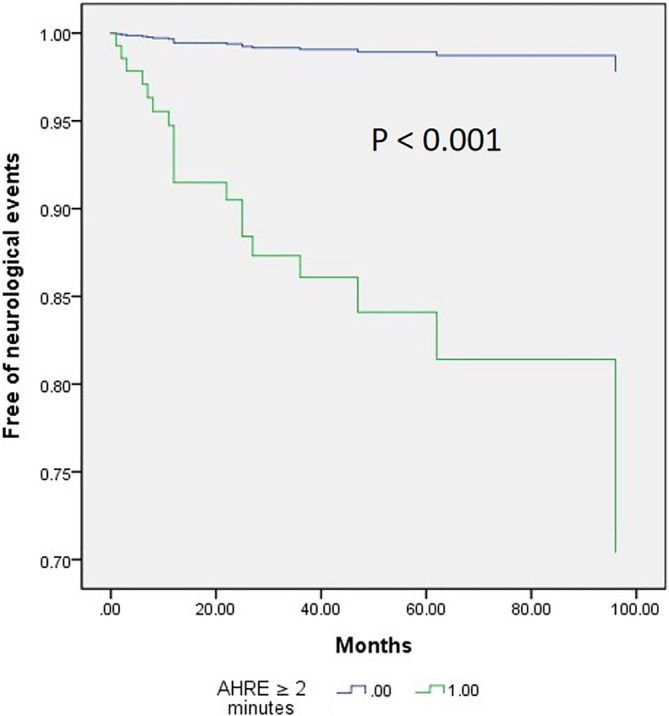
Cox regression event‐free survival curves from neurological events at 42.1 ± 31.2 months of follow‐up based on atrial high‐rate episode (AHRE) ≥2 min or not

The optimal AHRE cutoff value for association with future neurological events was determined to be 2 min, with the highest Youden index of 1.573 (sensitivity, 89.5%; specificity, 67.8%; positive predictive value, 13.6%; negative predictive value, 99.1%; positive likelihood ratio, 2.79; negative likelihood ratio, 0.15; AUC, 0.823; 95% CI, 0.763–0.884; p < 0.001) (Figure 1). With AHRE of 5 min, we found: sensitivity, 73.7%; specificity, 72.3%; positive predictive value, 13.1%; negative predictive value, 98.0%; positive likelihood ratio, 2.66; negative likelihood ratio, 0.38. Figure 2 shows the Cox regression event‐free survival curves for neurological events.

## 4 DISCUSSION

The main finding of this study is that AHRE duration ≥2 min, as detected by dual chamber PPMs, was significantly associated with neurological events in a Taiwanese population that had no history of AF. However, further investigation is warrant to confirm the current findings and to implement early aggressive anti‐thromboembolic therapy to prevent future neurological events based on detection of AHRE ≥2 min in Taiwanese population.

The ASSERT study^15^ is the only large, prospective trial to date to assess the relationship between AHRE (defined as an atrial rate of at least 190 beats/min lasting for ≥6 min) and systemic thromboembolic events in patients without a history of clinical AF. In the ASSERT study, stroke or systemic embolism occurred during follow‐up in 4.2% (1.7%/year) of patients in whom AHRE had been detected.^15^ In our study, stroke or TIA occurred during follow‐up in 5.3% (1.53%/year) of patients. The MOde Selection Trial, in which AHRE was defined as an atrial rate > 220 beats/min lasting ≥5 min,^6^ showed that patients with sinus node dysfunction in which AHRE was detected by pacemakers were more than twice as likely to die or have a stroke. A recent study showed that AHRE lasting ≥30 sec is a risk factor indicative of embolic stroke in a Japanese population with CIEDs.^5^ AHRE lasting ≥30 s is the shortest cutoff point determined in studies thus far; however, AUC = 0.67 in the Japanese study^5^ is relatively small compared to our result (AUC = 0.82).

In our study, the ROC curve showed that the best cutoff duration time of AHRE for predicting the risk of neurological events was 2 min. Compared to 5 min, our results showed that the cutoff value of 2 min had a higher positive likelihood ratio and negative predictive value, and lower negative likelihood ratio, indicating that 2 min is a more sensitive cutoff value for ruling out subsequent neurological events. Current guidelines^1^ recommend that AF be diagnosed using a 12‐lead EKG for a duration of more than 30 s. Both artifacts and false detection of far‐field R‐wave by the atrial lead could misclassify AHRE if of too short a duration. Previously, the 5 min cutoff value excluded most episodes of over‐sensing due to mechanical problems and appropriately detected clinical AF.^16^ In order to prevent over‐diagnosing SCAF we should focus on SCAF detected using our optimal cutoff value of AHRE ≥2 min confirmed by experienced electrophysiologists. Although both AHRE duration **≥** 6 h and AHRE duration **≥** 24 h are significantly different in patients with or without neurologic events in Table 1, however, in our multivariate analysis in Table 6, neither AHRE duration **≥** 6 h nor AHRE duration **≥** 24 h was independent predictor for neurological events. It may be related to relative small numbers of neurologic events in patients with AHRE duration **≥** 6 h (6, 10.9%) and AHRE duration **≥** 24 h (5, 13.5%) in Table 5, which were all less than AHRE duration **≥** 2 min (17, 13.6%).

Independent predictors for neurological events in our study were not only AHRE ≥2 min but also CHA_2_DS_2_‐VASc score. An increase in AHRE incidence with increasing CHA_2_DS_2_‐VASc score has been documented. The association was stronger with AHRE of increased duration, with CHA_2_DS_2_‐VASc demonstrating moderate accuracy as a predictor.^17^ All patients with neurological events had AHRE ≥2 min, except for two patients with AHRE ≥1 min. CHA_2_DS_2_‐VASc scores for all patients were 3 and HAS‐BLED scores were all 2. The 2020 European Society of Cardiology Guidelines recommend that, prior to initiating oral anticoagulation therapy, patients with AHRE >5–6 min have further electrocardiogram monitoring to document overt AF.^1^ The European Heart Rhythm Association, in a broadly endorsed 2017 consensus document regarding device‐detected AHRE, states that oral anticoagulation is recommended for patients with two additional risk factors: CHA2DS2‐VASc ≥2 in men, or ≥ 3 in women, and with AHRE burden >5.5 h/day.^18^

Based on our results, we suggest that patients with dual chamber PPMs in Taiwan, with documented AHRE ≥2 min following dual chamber pacemaker implantation, or AHRE ≥1 min and CHA_2_DS_2_‐VASc score ≥ 3, be considered for prescribed anticoagulants for stroke prevention.

Two large‐scale randomized clinical trials of non‐vitamin K oral anticoagulant for patients with device‐detected AHRE are ongoing.^19^, ^20^ The results may help illuminate the critical role of AHRE in stroke prevention.

## 5 STUDY LIMITATIONS

The present study has several limitations. First, this study has a single‐center, retrospective, observational design with a relatively small number of patients with dual chamber PPM in a hospital‐based setting, with all patients being Taiwanese. As a result, causality as a general conclusion for other populations, cannot be stated between AHRE and neurological events, since results may have been affected by the stated confounding factors. Second, AHRE may have been underestimated due to different default settings for AHRE in devices designed by different companies. Prospective multicenter studies with larger samples are required to confirm results of the present study. Third, this study did not reach any conclusions about the nature of heart rhythms at the time of the onset of stroke or TIA. Fourth, not all patients with neurological events underwent brain magnetic resonance imaging/angiography to pursue the etiologies of embolic origin, however, the neurologists confirmed the all neurologic events. Finally, the number of neurological outcomes is relatively small; therefore, there is a problem of over‐fitting with the multivariable analyses.

## 6 CONCLUSIONS

Stroke or TIA events are relatively common in Taiwanese patients with dual chamber PPMs. AHRE lasting for ≥2 min is an independent risk factor for neurological events in this population. AHRE of different durations appear to be consistently associated with neurological events. When AHRE ≥2 min is detected in patients with dual chamber PPMs, a comprehensive assessment of the risks and benefits of prescribing an anticoagulant should be considered.

## CONFLICT OF INTEREST

The authors declare no conflict of interest.

## AUTHOR CONTRIBUTIONS

Conception and design: Ju‐Yi Chen; data acquisition: Wei‐Da Lu, Ju‐Yi Chen; data analysis and interpretation: Wei‐Da Lu, Ju‐Yi Chen; statistical analysis: Wei‐Da Lu, Ju‐Yi Chen; drafting and finalizing the article: Ju‐Yi Chen; critical revision of the article for important intellectual content: Ju‐Yi Chen.

## Supporting information


**Figure S1** 

**Supplementary Table 1** Multivariate Cox regression for neurological events

## Data Availability

The data that support the findings of this study are available from Ju‐Yi Chen, MD, PhD.
