# Supplementary figures and images for: The optimal cutoff of atrial high‐rate episodes for neurological events in patients with dual chamber permanent pacemakers
Source: Clin Cardiol. 2021 May 18;44(6):871–9. doi: 10.1002/clc.23626 (PMC8207987; doi:10.1002/clc.23626)

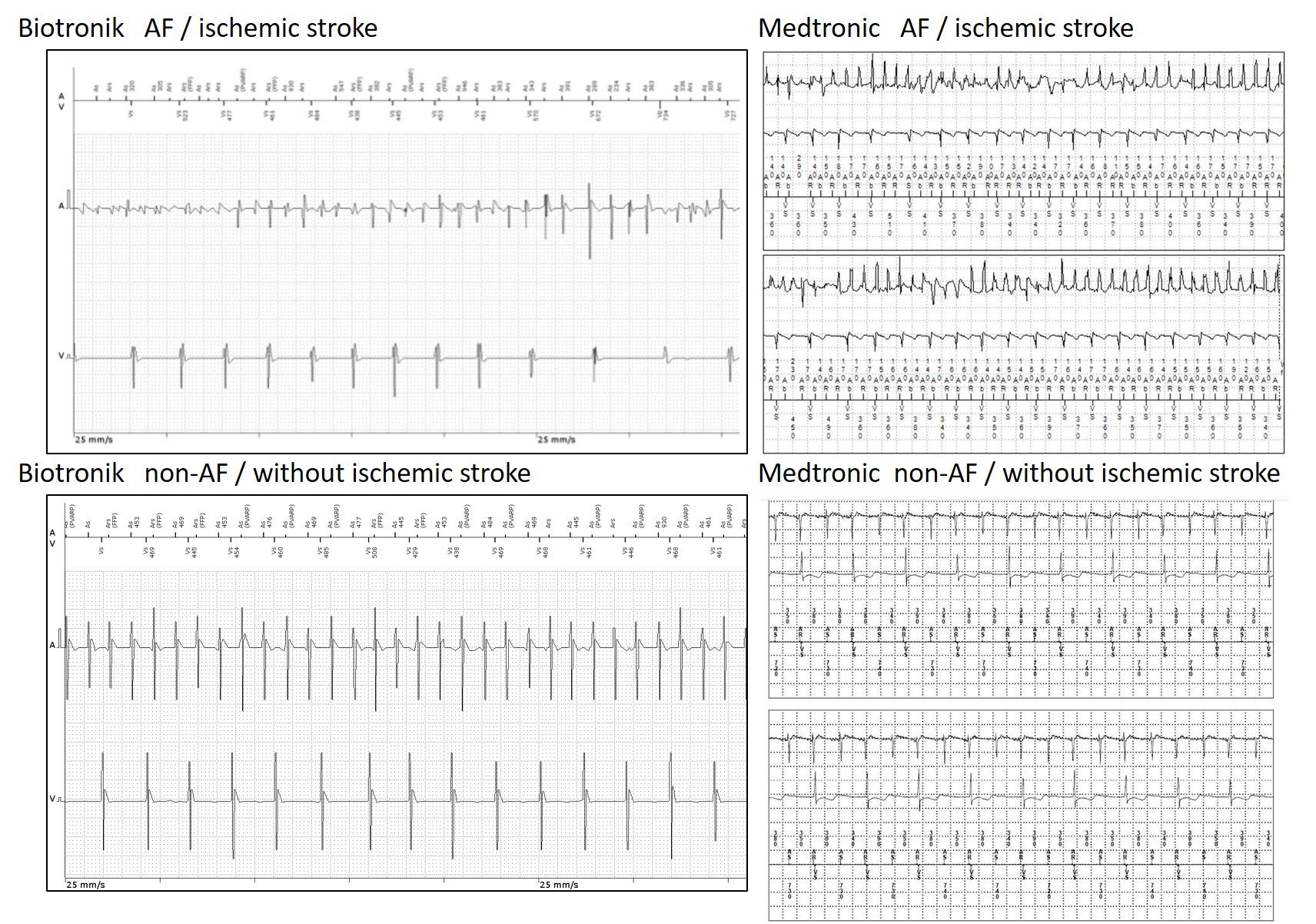

Supplement: Supplementary file 1 — Figure S1 [file CLC-44-871-s002.jpg]
