# Supplementary material for: The optimal cutoff of atrial high‐rate episodes for neurological events in patients with dual chamber permanent pacemakers
Source: Clin Cardiol. 2021 May 18;44(6):871–9. doi: 10.1002/clc.23626 (PMC8207987; doi:10.1002/clc.23626)
Supplement: Supplementary file 2 — Supplementary Table 1 Multivariate Cox regression for neurological events [file CLC-44-871-s001.docx]

| **Supplementary Table 1. Multivariate Cox regression for neurological events** | | | | | | | | | | | | | | | | | | |
| --- | --- | --- | --- | --- | --- | --- | --- | --- | --- | --- | --- | --- | --- | --- | --- | --- | --- | --- |
| Variables | **Multivariate Cox regression** | | | | | | | | | | | | | | | | | |
|  | Model 1 | | | Model 2 | | | Model 3 | | | Model 4 | | | Model 5 | | | Model 6 | | |
|  | **HR** | **95%CI** | ***p*** | **HR** | **95%CI** | ***p*** | **HR** | **95%CI** | ***p*** | **HR** | **95%CI** | ***p*** | **HR** | **95%CI** | ***p*** | **HR** | **95%CI** | ***p*** |
| Age (years) | 1.004 | 0.962-1.048 | 0.869 | 1.004 | 0.963-1.046 | 0.862 | 1.006 | 0.965-1.050 | 0.766 | 1.013 | 0.970-1.058 | 0.555 | 1.004 | 0.960-1.049 | 0.874 | 1.003 | 0.960-1.048 | 0.901 |
| Gender (female) | 1.591 | 0.519-4.875 | 0.417 | 1.586 | 0.518-4.860 | 0.419 | 1.731 | 0.563-5.329 | 0.339 | 1.591 | 0.507-4.990 | 0.426 | 2.144 | 0.695-6.607 | 0.184 | 2.202 | 0.717-6.756 | 0.168 |
| Diabetes mellitus | 2.896 | 0.979-8.564 | 0.055 | 2.797 | 0.945-8.278 | 0.063 | 2.934 | 1.010-8.528 | 0.048 | 2.991 | 1.042-8.583 | 0.042 | 3.087 | 1.068-8.919 | 0.037 | 3.094 | 1.074-8.912 | 0.036 |
| Prior stroke | 13.153 | 3.999-43.257 | <0.001 | 11.678 | 3.552-38.392 | <0.001 | 8.911 | 2.777-28.593 | <0.001 | 9.337 | 2.968-29.377 | <0.001 | 10.015 | 3.075-32.614 | <0.001 | 9.716 | 3.021-31.243 | <0.001 |
| Device (MEDTRONIC) | 1.131 | 0.305-4.188 | 0.854 | 1.075 | 0.102-1.298 | 0.119 | 0.682 | 0.181-2.571 | 0.572 | 0.465 | 0.127-1.705 | 0.248 | 0.364 | 0.102-1.298 | 0.119 | 0.380 | 0.106-1.362 | 0.137 |
| AHRE Duration**≥**30secs | 323650 | 0.000-1328 | 0.911 |  |  |  |  |  |  |  |  |  |  |  |  |  |  |  |
| AHRE Duration**≥**1mins |  |  |  | 357636 | 0.000-1229 | 0.910 |  |  |  |  |  |  |  |  |  |  |  |  |
| AHRE Duration**≥**2mins |  |  |  |  |  |  | 13.406 | 2.959-60.743 | 0.001 |  |  |  |  |  |  |  |  |  |
| AHRE Duration**≥**5mins |  |  |  |  |  |  |  |  |  | 5.725 | 1.960-16.720 | 0.001 |  |  |  |  |  |  |
| AHRE Duration**≥**6hrs |  |  |  |  |  |  |  |  |  |  |  |  | 2.401 | 0.862-6.687 | 0.094 |  |  |  |
| AHRE Duration**≥**24hrs |  |  |  |  |  |  |  |  |  |  |  |  |  |  |  | 2.950 | 1.008-8.634 | 0.048 |
| Data are presented as mean±SD or n (%).  Abbreviations: AHRE: atrial high-rate episodes. | | | | | | | | | | | | | | | | | | |
